# Initiation of Electron Transport Chain Activity in the Embryonic Heart Coincides with the Activation of Mitochondrial Complex 1 and the Formation of Supercomplexes

**DOI:** 10.1371/journal.pone.0113330

**Published:** 2014-11-26

**Authors:** Gisela Beutner, Roman A. Eliseev, George A. Porter

**Affiliations:** 1 University of Rochester Medical Center, Department of Pediatrics, Division of Cardiology, 601 Elmwood Ave., Box 631, Rochester, New York 14642, United States of America; 2 Center for Musculoskeletal Research, University of Rochester, 601 Elmwood Ave., Rochester, New York 14642, United States of America; National Institute of Environmental Health Sciences, United States of America

## Abstract

Mitochondria provide energy in form of ATP in eukaryotic cells. However, it is not known when, during embryonic cardiac development, mitochondria become able to fulfill this function. To assess this, we measured mitochondrial oxygen consumption and the activity of the complexes (Cx) 1 and 2 of the electron transport chain (ETC) and used immunoprecipitation to follow the generation of mitochondrial supercomplexes. We show that in the heart of mouse embryos at embryonic day (E) 9.5, mitochondrial ETC activity and oxidative phosphorylation (OXPHOS) are not coupled, even though the complexes are present. We show that Cx-1 of the ETC is able to accept electrons from the Krebs cycle, but enzyme assays that specifically measure electron flow to ubiquinone or Cx-3 show no activity at this early embryonic stage. At E11.5, mitochondria appear functionally more mature; ETC activity and OXPHOS are coupled and respond to ETC inhibitors. In addition, the assembly of highly efficient respiratory supercomplexes containing Cx-1, -3, and -4, ubiquinone, and cytochrome *c* begins at E11.5, the exact time when Cx-1 becomes functional activated. At E13.5, ETC activity and OXPHOS of embryonic heart mitochondria are indistinguishable from adult mitochondria. In summary, our data suggest that between E9.5 and E11.5 dramatic changes occur in the mitochondria of the embryonic heart, which result in an increase in OXPHOS due to the activation of complex 1 and the formation of supercomplexes.

## Introduction

The heart is the first functional organ in the vertebrate embryo. In the mouse, the heart begins to beat at about embryonic day (E) 8.25, and cardiac formation is largely complete by E14, the end of the embryonic period [1]. Survival of the embryo depends upon intact cardiac function after about E10, at around the time the placenta becomes active. Over the remainder of gestation and immediately after birth, the functional heart remodels, such that it begins to reach its cellular and organ structural maturity at about a week after birth [1].

The adult heart generates most of its needed energy in the form of ATP by a combination of β-oxidation and oxidative phosphorylation (OXPHOS) in the mitochondria. In contrast, the early embryonic heart is thought to generate ATP exclusively by anaerobic glycolysis due to the low supply of oxygen. During the late embryonic period, a transition to aerobic glycolysis and lactate oxidation occurs, which is followed by the switch to β-oxidation after birth (reviewed in [1]–[4]). It is worth noting that these ideas about bioenergetics during embryonic cardiac development are largely inferred from older studies that have not been validated using modern techniques.

Many studies suggest that mitochondrial function is important for development. For example mutation of mitochondrial proteins often cause embryonic demise [4], [5]. In addition, we recently showed that changes in bioenergetics control myocyte differentiation in the embryonic heart [6], and similar changes in mitochondrial function play a role in stem cell differentiation [7]. However, it remains unclear how and when during cardiac development mitochondria begin to perform their primary function of producing energy. In particular, the activity of the ETC and its role in regulating myocyte differentiation in the early heart remain unknown, despite its obvious importance in the mature heart.

Generation of ATP by OXPHOS requires the production of NADH and FADH_2_ in the Krebs cycle and a functional ETC. The four protein complexes of the ETC are located in the cristae membrane and complexes (Cx) -1, -3, and -4 are organized in respiratory supercomplexes called respirasomes [8]–[10]. Respirasomes increase the efficiency of electron transport and the generation of the proton motive force (Δµ_H_ = the electrical gradient (Δψ_m_) + the pH gradient (Δ_pH_)) across the inner mitochondrial membrane (IMM) that is used by Cx-5 (ATP synthase) to make ATP. The factors that drive the formation of supercomplexes are unknown, but Cx-1 assembly and activation is critical for this process [11].

In this study, we explored activation of the ETC in the embryonic heart by adapting biochemical/mitochondrial techniques for use with limited amounts of embryonic tissues. We show that the ETC becomes increasingly active in the heart during the embryonic period, and that this is due to the activation of Cx-1 and the initiation of respiratory supercomplex assembly.

## Experimental Procedures

### Ethics

Procedures were in strict accordance with the Division of Laboratory Animal Medicine, University of Rochester, in compliance with state law, federal statute, and NIH policy and were approved by the Institutional Animal Care and Use Committee of the University of Rochester (University Committee on Animal Resources (UCAR) protocol 2011-003).

### Animals

All experiments were performed using wild type mice in a C57BL/6N background [6], [12], [13]. Mice were sacrificed by cervical dislocation and the embryos were harvested from the mother immediately. Embryos were harvested on E9.5, E11.5 and E13.5 based on timed mating and the embryo morphology. All experiments used tissue from the left and right ventricles and outflow tract to limit contamination by atrial myocytes.

### Tissue preparation

Mitochondria from adult and E13.5 mouse hearts were isolated using a published protocol [14].

Tissue homogenates from mouse embryonic and adult hearts were obtained by homogenizing tissue in approximately 0.1 ml ice-cold mannitol/sucrose/Tris buffer in Eppendorf tubes fitted with a Teflon pestle. The tissue homogenates were kept on ice and used within 30 minutes of preparation. Tissue homogenates used for native or denaturing electrophoresis were prepared from embryonic hearts with a Dounce glass homogenizer in ice-cold buffer consisting of 440 mM sucrose, 20 mM MOPS (pH 7.2), 1 mM EDTA and protease inhibitor cocktail. The protein concentrations were determined with a BCA kit (BioRad).

### Determination of mitochondrial oxygen consumption and the respiratory control ratio (RCR)

Oxygen consumption of isolated heart mitochondria or tissue homogenates was measured with a Clark type oxygen electrode (Hansatech, PP Systems, Boston MA) using published protocols [14], [15]. Measurements were carried out at room temperature in 0.3 ml of respiration medium (70 mM mannitol, 25 mM sucrose, 20 mM HEPES, 120 mM KCl, 5 mM KH_2_PO_4_, 3 mM MgCl_2,_ pH 7.4). Substrate mediated respiration (state 2 [16] or V_0_) was initiated by the addition of 3 mM malate and 5 mM glutamate or the addition of 2 mM succinate. Then, 1 mM ADP was added to obtain maximal respiration (state 3 [16] or V_max_). RCR was calculated as the ratio of V_max_ over V_0_ [14], [17]. 10–100 µM atractyloside (ATR) was added at the end of each experiment to test IMM coupling. Alternatively, 100 µM (as increments of 20 µM or a single addition) potassium cyanide (KCN) was added to assess Cx-4 function.

### Activity tests for complex-1 and -2 and citrate synthase

The activity of Cx-1, Cx-2 and the citrate synthase was measured with a Genesys 5 UV/Vis spectrophotometer (Thermo Scientific, Pittsburgh PA) at room temperature using published protocols [18], [19]. The spectrophotometrically obtained change of absorbance per minute (ΔE) was used to calculate the activity of enzymes (Units) according to the law of Beer and Lambert. ([20], chapter 4, page 168). The enzymatic activity was then normalized to mg protein, so that the activity is expressed as Units/mg (U/mg) or milli units/mg (mU/mg). Generally, the test-volume was 0.5 ml, and 1–5 µg of protein from a cardiac tissue homogenate or isolated mitochondria was used. Samples were frozen and thawed twice prior to testing.

Cx-1 activity was measured as NADH-ubiquinone oxidoreductase (340 nm; ε for NADH 6.81 mM^−1^cm^−1^), NADH-cytochrome *c* reductase (550 nm, ε for cytochrome *c* = 18.7 mM^−1^cm^−1^), or NADH-ferricyanode reductase ( = NADH oxidase; 420 nm, ε for ferricyanide = 1 mM^−1^cm^−1^) activity. Where necessary, the test was repeated with 2 µg/ml rotenone to assess mitochondria specific, rotenone-sensitive activity. NADH oxidase activity of tissue homogenates (5 µg) was also measured using the Complex I Enzyme Activity Dipstick Assay (Abcam, Cambridge, MA).

Active and deactive forms of Cx-1 were accessed by incubating tissue homogenates with 1 mM N-ethyl-maleimide (NEM) in 0.25 M sucrose, 50 mM Tris-HCl, 0.2 mM EDTA pH 7.0 for 10 minutes. The Cx-1 activity was then measured using the NADH-ferricyanide reductase assay. In the absence of NEM this test shows after the addition of 0.1 mM NADH into the cuvette a first phase with a high initial activity due to activation of NADH oxidases and deactive Cx-1. This phase of high activity transitions into a second, longer lasting phase with lower activity [19], indicative of active Cx-1. In the presence of NEM, the initial high activity was less or absent in embryonic samples and only the second phase with very low activity remained. Since NEM inhibits only the deactive form of Cx-1, the difference of the initial activity in the absence and presence of NEM is considered the de-active form of Cx-1.

Cx-2 (succinate dehydrogenase) was measured using the conversion of succinate to fumarate, and phenazin methosulfate is used as an artificial electron acceptor to reduce cytochrome *c* [21]. KCN (2 mM) was included to prevent reoxidation of cytochrome *c* by Cx-4. The change of absorbance is measured at 550 nm and ε is 18.7 mM^−1^×min^−1^.

Citrate synthase activity was measured using the conversion of acetyl CoA and oxaloacetate to citrate and CoA-SH, which reacts with 5,5-dithiobis-(2-nitrobenzoic acid) to form 5-thio-2-nitrobenzoate to increase of absorbance at 412 nm [21].

In-gel assay for Cx-1 were performed as published [22]. Briefly, hrCN gels or stripes were placed in assay solution containing 5 mM tris, pH 7.4 and 2.5 mg/mL nitroblue tetrazolium and 0.1 mg/mL NADH. The gel was developed and fixed (50% methanol, 10% acetic acid).

### Complex 3 immunocapture

To immunocapture Cx-3, cardiac tissue homogenates (samples of 50–100 µg protein) were diluted with phosphate buffered saline to 0.25 µg/µl, protease inhibitor cocktail (Halt, Pierce) was added, and the samples were solubilized on ice with lauryl-maltoside (final concentration 1 mM). After centrifugation to remove tissue fragments, mitochondrial supercomplexes were immunocaptured with a Cx-3 specific monoclonal antibody (clone 11A51H12, dilution 1: 100, ab109862 (Abcam) and precipitated with protein G agarose (Roche Bioscience). Proteins were eluted from the beads by adding Laemmli sample buffer.

### SDS and high-resolution clear native (hrCN) electrophoresis

Denaturing and native electrophoresis was done according to published protocols [22]–[24]. Denatured proteins were separated on 16% SDS gels. For hrCN electrophoresis, protein complexes were separated on clear-native gradient gels (4–16%). To isolate protein complexes from tissue homogenates, samples were resuspended in extraction buffer (50 mM NaCl, 50 mM Imidazole (pH 7 at 4°C), 2 mM aminocaproic acid and 1 mM EDTA). The tissue homogenate was lysed with 4 g digitonin/g protein for 20 minutes on ice. The separated proteins/protein complexes were transferred onto nitrocellulose membranes, which were incubated with the primary antibody and horseradish peroxidase coupled secondary antibody, and labeling was detected by enhanced chemiluminescence (GE Healthcare, Piscataway NJ). The following antibodies were used: mouse anti-beta-actin (sc-47778, Santa Cruz), mouse anti-NDUFA9 (Abcam ab14713), rabbit anti-NDUFAB1 (Abcam ab96230), mouse anti-NDUFB6 (Abcam ab110244), rabbit anti-Cox 4 (Thermo Fisher P-29992), rabbit anti ubiquinone-cytochrome c dehydrogenase core protein 1 precursor (Thermo Fisher PA5-21394), and rabbit anti-VDAC (Abcam 34726).

In some cases, immunoblots were analyzed for band density using Image J. Background density was subtracted from band density. Data from SDS immunoblots (VDAC) were normalized to beta-actin.

### Mitochondrial DNA Quantitation

Total cellular DNA was isolated using the Wizard SV DNA purification kit (Promega), according to the manufacturer’s instructions. One hundred ng of total DNA per sample was subjected to real-time quantitative PCR analysis using SYBR Green reagent (Quanta) and RotorGene real-time DNA amplification system (Qiagen). To detect mitochondrial DNA (mtDNA) levels, we used a primer pair for the mtDNA-encoded gene, CO1 (5′-GCC CCA GAT ATA GCA TTC CC-3′ and 5′-GTT CAT CCT GTT CCT GCT CC-3′). To detect genomic DNA (gDNA) levels, we used a primer pair for the nuclear-encoded 18s (5′-TAG AGG GAC AAG TGG CGT TC-3′ and 5′-CGC TGA GCC AGT CAG TGT- 3′). MtDNA was normalized to gDNA.

### Analysis and statistics

Data sets used for statistical analysis represent at least n = 3 and all data are presented ± SE. After testing for normality of the data, significance was determined for normal data by using Student T-test or one-way ANOVA for multiple groups with post-hoc testing and for non-normal data using Friedman’s with post-hoc testing using Prism (V6, GraphPad, La Jolla, CA). Significance is defined as p≤0.05, and only significant differences are noted in the figures.

## Results

### Electron transport chain activity increases during embryonic cardiac development

We recently showed that mitochondrial structure in cardiac myocytes becomes more complex and the mitochondrial membrane potential (Δψ_m_) increases as the heart develops leading to a drop in mitochondrial-derived oxidative stress that enhanced myocyte differentiation [6]. Interestingly, inhibiting Cx-1 of the ETC with rotenone had no effect on Δψ_m_ at E9.5, as it did at older ages, while inhibiting Cx-2 with malonate decreased Δψ_m_ at all ages [6]. These data suggested that ETC function changes during cardiac development, and this change is the focus of this study.

#### Mitochondrial oxygen consumption

We examined ETC function by measuring oxygen consumption of heart tissue homogenates from E9.5, E11.5 and E13.5 embryos and adult mice using a Clark electrode. Since at E9.5 and E11.5 embryos are very small, it was not feasible to isolate mitochondria from them. On average, a single heart of an E9.5 embryo provides 1.64±0.09 µg protein. Therefore, for each of these experiments we pooled 7–8 E9.5 hearts (12.58±1.54 µg protein; n = 9 experiments). At E11.5 a single heart provides on average 19.79±1.08 µg protein, and 2–3 E11.5 hearts (42.21±9.86 µg protein, n = 15 experiments) where used per experiment. An average E13.5 heart provides 55.43±7.66 µg protein (n = 9 experiments), and 1 heart per experiment was used. Adult samples contained on average 100 µg protein. Some experiments were performed in the presence of the Cx-1, Cx-2, Cx-4, and adenine nucleotide translocase (ANT) inhibitors rotenone, malonate, potassium cyanide (KCN), and ATR, respectively.

**Figure 1:**
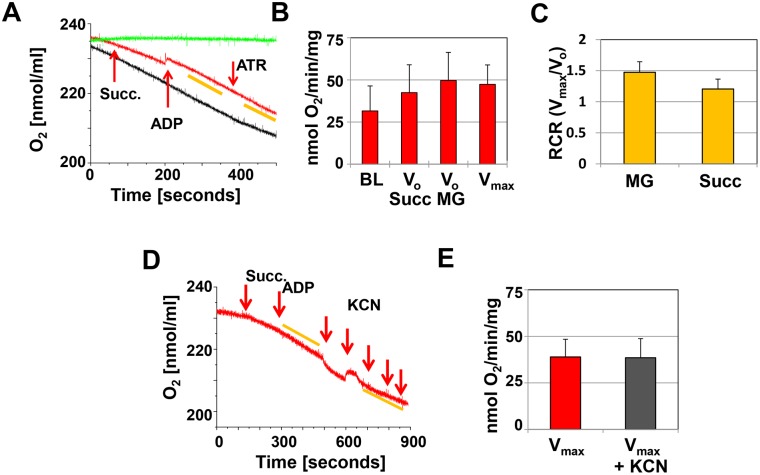
Oxygen consumption at E9.5. **A.** Representative recording of oxygen consumption in cardiac tissue homogenates (approximately 12.5 µg protein in 0.3 ml respiration buffer). The red recording represents the experiment and the arrows indicate the addition of 2 mM succinate (Succ.), 1 mM ADP and 0.1 mM ATR. The yellow bars indicate the slopes used to calculate oxygen consumption. The black recording represents the tissue homogenate in the respiration medium, but no additions to stimulate oxygen consumption are made. The green recording represents the buffer only. **B.** Bar graph illustrating oxygen consumption at baseline (BL), after the addition of substrate (V_o_ Succ and V_o_ MG) and ADP (V_max_). **C.** Respiratory control ratio, calculated as V_max_/V_o_. A–C: n = 9, for each experiment 7–8 hearts were pooled. **D.** Representative recording of the inhibition of oxygen consumption by potassium cyanide (KCN) added in 20 µM increments. **E.** Summary of KCN inhibition (n = 3, 9–10 hearts were pooled per experiment).

We recorded the consumption of oxygen at baseline (BL) with only the homogenate in the respiration medium, after the addition of the Cx-1 substrates malate/glutamate (NADH generating) or the Cx-2 substrate succinate (FADH generating) to obtain V_0_, and after the addition of ADP to obtain V_max_. There was significant oxygen consumption in E9.5 hearts at baseline, and this did not change significantly over time or after the addition of succinate, malate/glutamate, or ADP (Figure 1A, red and black recordings, and Figure 1B). Oxygen consumption that does not respond to any activators or inhibitors of the ETC may indicate that the IMM is leaky or uncoupled or that other oxidases use the oxygen in the buffer [25], so we tested both possibilities. The respiratory control ratio (RCR) of V_max_/V_0_, approaches 1 when the IMM is uncoupled, and we found this to be the case in E9.5 homogenates in the presence of either Cx-1 or Cx-2 substrates (Figure 1C). In addition, the rate of oxygen consumption remained the same after the addition of ATR or the addition of the Cx-4 inhibitor KCN (Figure 1A, D, and E, for further information on ATR, see below), indicating that the oxygen consumption was not due to ETC activity in E9.5 embryonic hearts.

**Figure 2:**
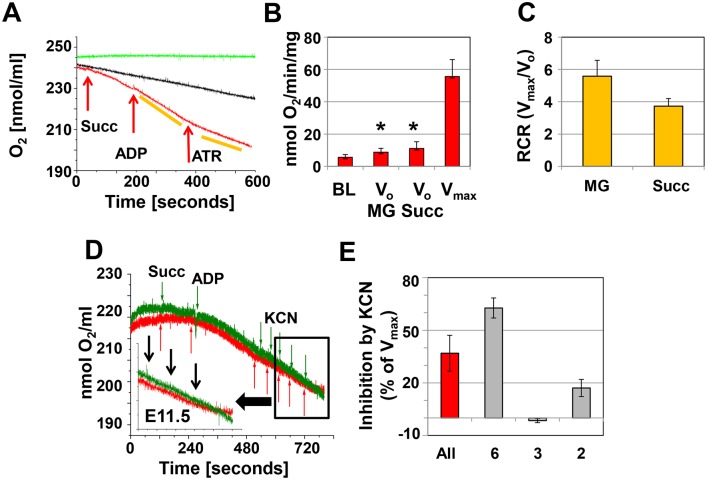
Oxygen consumption at E11.5. **A.** Representative recording of oxygen consumption in cardiac tissue homogenates (approximately 20 µg protein in 0.3 ml respiration buffer). The red recordings represents the experiment and the arrows indicate the addition of 2 mM succinate (Succ.), 1 mM ADP and 0.1 mM ATR. The yellow bars indicate the slopes used to calculate oxygen consumption. The black recording represents the tissue homogenate in the respiration medium, but no additions to stimulate oxygen consumption are made. The green recording represents the buffer only. **B.** Bar graph illustrating oxygen consumption at baseline (BL), V_o_ (malate/glutamate (MG) and Succ), and V_max_. *indicates significance (p≤0.001, ANOVA V_o_ compared to V_max_). **C.** Respiratory control ratio, calculated as V_max_/V_o_. **A–C:** n = 14 experiments, for each experiment 2–3 hearts were pooled. **D.** Representative recordings showing variable inhibition of oxygen consumption by potassium cyanide (KCN) added in 20 µM increments. Red recording: KCN is inhibitory, green recording: no inhibitory effect. **E.** Summary of KCN inhibition (n = 11; 3–4 hearts were pooled per experiment). All represents the composite data of all experiments, whereas the other columns represent data pooled from experiments with similar results.

In contrast, homogenates from E11.5 hearts responded distinctly to the addition of malate/glutamate, succinate, and ADP (Figure 2A and B). At this embryonic stage, V_max_ was not different than that seen in E9.5 hearts, regardless of the substrate. However, V_0_ was lower than V_max_ at E11.5 (Figure 2A and B) and V_0_ at E9.5 (p≤0.007; n = 15, T-test), increasing the RCR to 5.58±0.98 with malate/glutamate as substrate and 3.72±0.47 with succinate as substrate (Figure 2C). The increased RCR indicates that mitochondria in E11.5 hearts are more mature, have the capacity for substrate oxidation and ATP turnover, and exhibit coupling of electron transport and ATP synthesis. Furthermore, KCN effectively inhibited V_max_ in most experiments by more than 60%, while there was little or no inhibition in the remaining samples (Figure 2D and E). Variable effects on oxygen consumption were also observed with ATR (see below).

**Figure 3:**
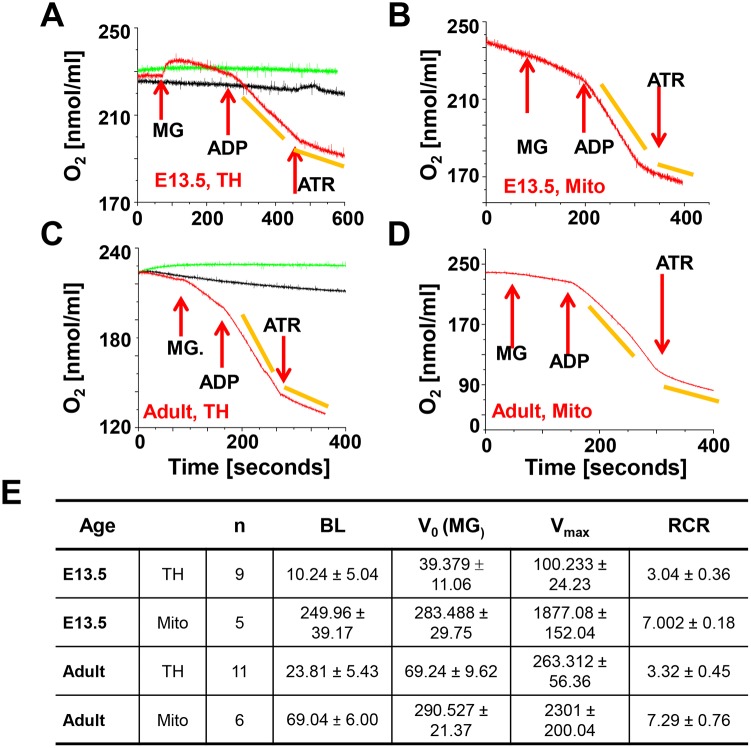
Oxygen consumption at E13.5 and in adults. **A and B.** Tissue homogenate (TH, approximately 55 µg protein) and isolated mitochondria (Mito) from E13.5 embryos. Hearts of 31 embryos were used to isolate 30 µg of mitochondrial protein for this experiment. **C and D.** Tissue homogenate (TH, adult) and isolated mitochondria (Mito, adult). 100 µg protein were used per experiment. In all panels the red recordings represents the experiment and the arrows indicate the addition of 3 mM malate and 5 mM glutamate (MG), 1 mM ADP and 0.1 mM ATR. The yellow bars indicate the slopes used to calculate oxygen consumption. The black recording represents the tissue homogenate in the respiration medium, but no additions to stimulate oxygen consumption are made. The green recording represents the buffer only. **E.** Summary of V_0_, V_max_ and the RCR of E13.5 and adult samples, where n is the number of experiments, BL baseline, MG malate/glutamate, RCR respiratory control ratio. BL, V_o_ and V_max_ are calculated as nmol O_2_/min/mg.

**Figure 4:**
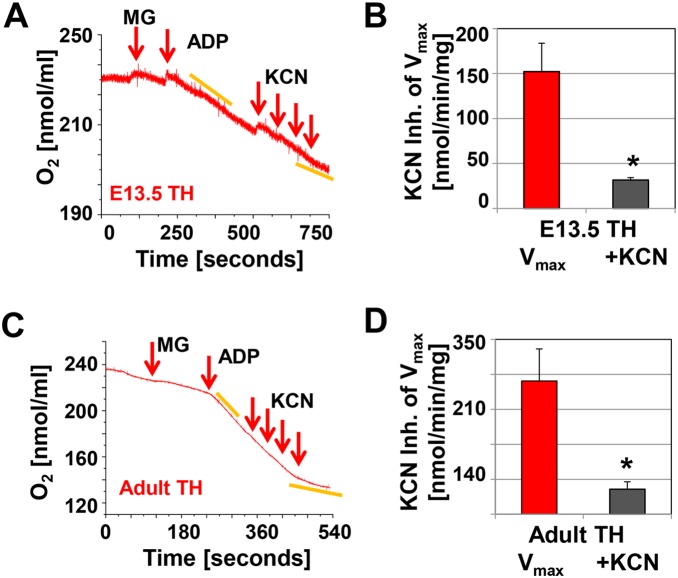
Inhibition of oxygen consumption by KCN at E13.5 and in adult mice. **A and C.** Representative recordings of KCN inhibited oxygen consumption in cardiac tissue homogenates (E13.5 approximately 55 µg protein in 0.3 ml respiration buffer; adult 100 µg protein). The arrows indicate the addition of 3 mM malate and 5 mM glutamate (MG), 1 mM ADP and the addition of KCN (increments of 20 µM). **C and D.** Bar graphs, showing the inhibition of V_max_ in tissue homogenates (TH) by KCN; n = 4, *indicates significance, p≤0.001, T-test.

In homogenates of E13.5 hearts, V_0_ was about 40 pmol/min/µg with malate/glutamate, while V_max_ was 100.23±22.99 pmol/min/µg and the RCR was 3.04±0.36 (Figure 3A and 3E), which was lower than that observed in E11.5 hearts. However, a lower RCR of 3.32±0.45 (n = 11) was also measured in adult tissue homogenates (Figure 3E, suggesting that extra-mitochondrial factors (leaks, usage of cellular substrates) present in the tissue homogenates may have altered the measurements [26]. Therefore, we isolated mitochondria from E13.5 and adult mouse hearts (Figure 3B and 3D) and found that their V_0_, V_max_, and RCR values were not different from published values for adult heart mitochondria [14] and were not dependent on age (Figure 3E). The Cx-4 inhibitor KCN inhibited oxygen consumption in tissue homogenates and isolated mitochondria in all experiments (Figure 4A–D). In summary, in mitochondria from E13.5 hearts electron transport and ATP synthesis were coupled.

Next, to determine the roles of Cx-1 and -2 in ETC function in the embryonic heart, we investigated the effect of the inhibitors rotenone (Cx-1) and malonate (Cx-2) on oxygen consumption in tissue homogenates. As oxygen consumption was not due to the ETC activity at E9.5 (see above), we did not expect to see inhibition using rotenone and malonate at this age, and this was indeed the case in a limited number of experiments (Table 1). In E11.5 heart homogenates, the Cx-1 inhibitor rotenone inhibited malate/glutamate-mediated (n = 3), but not succinate-mediated, respiration, as expected. Interestingly, the Cx-2 inhibitor malonate inhibited both malate/glutamate- and succinate-mediated oxygen consumption (Table 1). Finally, in both E13.5 and in adult tissue homogenates, inhibition of Cx-1 prevented malate/glutamate-mediated, but not succinate-mediated, respiration, while inhibition of Cx-2 had no effect on malate/glutamate-mediated respiration but inhibited succinate-mediated respiration (Table 1). These data suggest that, by E13.5, Cx-1 and Cx-2 accept electrons from NADH and FADH_2_ and contribute to OXPHOS, but at earlier stages of cardiac development, these complexes are not fully functional.

**Table 1:** Effect of rotenone and malonate on oxygen consumption^1^.

	Inhibitor^2^	exp^3^	Substrate	Effect on Oxygen consumption^4^
**E9.5**	Rotenone	1	Malate/glutamate	NA
			Succinate	
**E9.5**	Malonate	1	Malate/glutamate	NA
			Succinate	
**E11.5**	Rotenone	3	Malate/glutamate	Inhibition
			Succinate	None
**E11.5**	Malonate	3	Malate/glutamate	Inhibition
			Succinate	Inhibition
**E13.5**	Rotenone	3	Malate/glutamate	Inhibition
			Succinate	None
**E13.5**	Malonate	3	Malate/glutamate	None
			Succinate	Inhibition
**Adult**	Rotenone	3	Malate/glutamate	Inhibition
			Succinate	None
**Adult**	Malonate	3	Malate/glutamate	None
			Succinate	Inhibition

1 All experiments were performed with cardiac tissue homogenates.

2 Concentrations used: 1 mM malonate, 2 µg/ml rotenone.

3 Number of individual experiments for each condition. E9.5: 7–9 hearts were pooled per experiment; E11.5 2–3 hearts were pooled per experiment.

4 NA–not applicable as Cx-1 and -2 substrates do not increase oxygen consumption at E9.5 and thus inhibitors have no effect.

#### E11.5 is a critical time point for ETC activity in mouse hearts

ATR locks the ANT in its cytoplasmic-side open conformation and thus inhibits the exchange of ATP and ADP across the IMM. As a result, adding ATR at the end of the oxygen consumption experiments provides a test of the integrity of the IMM, as it should inhibit all mitochondrial respiration unless the IMM is uncoupled [27]. However, ATR can also activate the mitochondrial permeability transition pore (mPTP) [28] and thus uncouple the IMM and increase futile oxygen consumption by hydrolyzing ATP to reestablish Δψ_m_ [29]. We found that ATR had no effect on the oxygen consumption rates in heart homogenates at E9.5 (Figure 1A), consistent with the hypothesis that the mitochondria at this stage are uncoupled. In contrast, ATR inhibited respiration in all E13.5 and adult heart samples (Figures 3A–D), suggesting that it inhibited the ANT.

**Figure 5:**
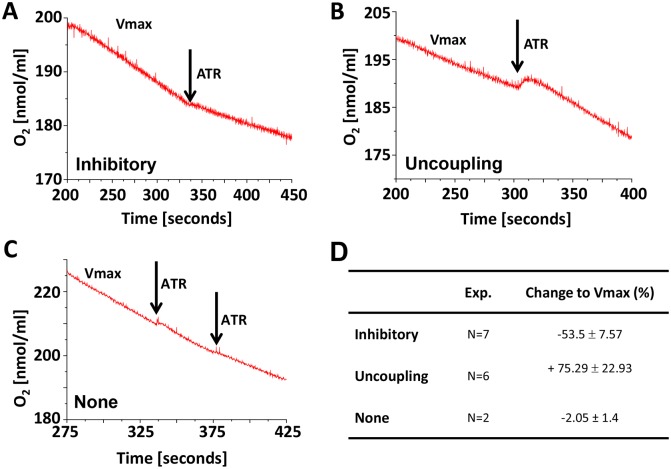
Variable effect of ATR on mitochondrial respiration at E11.5. **A–C.** Representative recordings of the effect of ATR on V_max_; **A.** inhibitory, **B.** uncoupling or **C.** none. **D.** Summary data of changes to V_max_ for each response.

However, in E11.5 homogenates, the addition of 100 µM ATR had variable effects. In about an equal number of experiments, ATR either inhibited ANT (Figure 5A) or uncoupled oxygen consumption (Figure 5B), while in a minority of experiments it had no effect (Figure 5C). Finally, in a limited number of experiments, we found that the uncoupler dinitrophenol uncoupled respiration when ATR inhibited OXPHOS but not when it increased oxygen consumption. Collectively, these data indicate that E11.5 represents a critical time to establish mitochondrial function: in some specimens mitochondrial oxygen consumption is inhibited by ATR, while in others, it had no effect or even increased oxygen consumption. Since the mPTP is open at E9.5 [6], we speculate that E11.5 represents a critical moment in establishing mitochondrial ETC activity, when the mPTP closes and IMM coupling becomes possible.

#### Complex-1 and -2 activity

**Figure 6:**
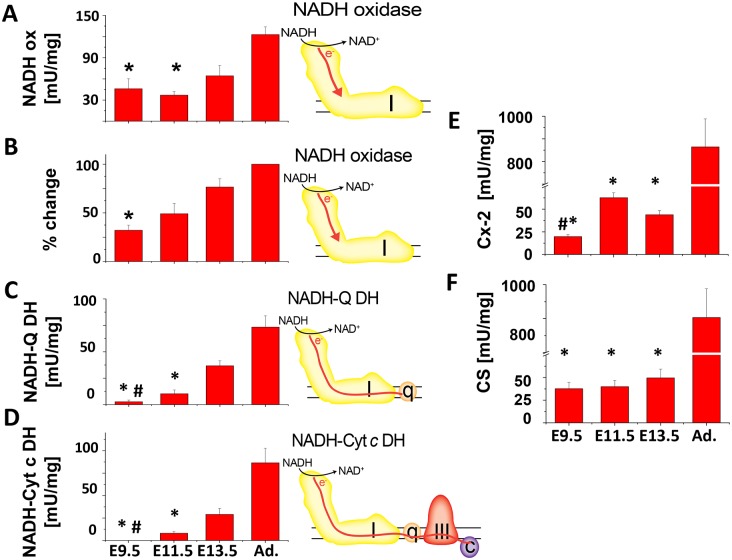
Enzymatic activity of Cx-1, Cx-2 and citrate synthase in cardiac tissue homogenates. Each assay was done with 1–5 µg protein of cardiac tissue homogenate. The activities are given in mU/mg, except for the dipstick assay, where the relative change was calculated from signal of adult homogenates (100%). In **A–D**, the cartoon accompanying each graph depicts the flow of electrons through Cx-1 (I) and -3 (III), ubiquinone (q), and cytochrome *c* (c) tested in the assay. **A.** NADH-oxidase (NADH ox) assay, *p≤0.05 E9.5 or E11.5 versus adult. **B.** Cx-1, NADH-oxidase (NADH ox) dipstick assay, *p≤0.05 E9.5 versus adult. **C.** NADH-ubiquinone dehydrogenase (NADH-Q-DH) assay, *p≤0.05, E 9.5 or E11.5 compared to older embryos or adults; #p≤0.05, E9.5 versus E11.5. **D.** NADH-cytochrome *c* dehydrogenase (NADH-Cyt c DH) assay, *p≤0.05, E 9.5 or E11.5 compared to older embryos or adults; #p≤0.05, E9.5 versus E11.5 **E.** Cx-2/succinate dehydrogenase assay, *p≤0.05, embryos compared to adults; #p≤0.05, E9.5 versus E11.5 and **F.** Citrate synthase assay. *p≤0.05, embryos compared to adults; #p≤0.05, E9.5 versus E11.5. In all experiments, ANOVA with Tukey post-hoc testing was used and n≥3. All other comparisons were not significant.

To further evaluate ETC function, we measured the activity of Cx-1 and Cx-2 using spectrophotometric-based enzyme activity assays on embryonic heart homogenates. Cx-1 activity was measured using different assays (see cartoons in Figure 6A–D): 1) NADH oxidoreductase activity was calculated from the total activity of the NADH-ubiquinone and NADH-cytochrome *c* oxidoreductase (below) in the samples prior the addition of rotenone. In addition, we used the ferricyanide oxidoreductase assay, which measures the total NADH oxidase activity in a sample but has the tendency to overestimate the cellular NADH oxidase activity. 2) The Cx-I immunocapture dipstick assay measures Cx-1-specific NADH oxidase activity, or the ability of Cx-1 to accept electrons from NADH. 3) The NADH-ubiquinone oxidoreductase assay measures the ability of electrons from NADH to be transferred to ubiquinone by Cx-1. 4) The NADH-cytochrome *c* oxidoreductase assay measures electron transfer from NADH through Cx-1 and ubiquinone to Cx-3.

Both the dipstick and the spectrophotometric (Figure 6A and B) assays showed that NADH oxidase activity was present in significant amounts in all embryonic stages and increased steadily as the heart matures. In contrast, NADH-ubiquinone oxidoreductase (Figure 6C) and NADH-cytochrome *c* oxidoreductase (Figure 6D) activities were virtually absent at E9.5, but increased at later embryonic stages. We also found measureable Cx-2 (succinate dehydrogenase) activity at E9.5 that increased during cardiac development but nevertheless remained much lower than that seen in adult heart homogenates (Figure 6E). In addition, the activity of the citrate synthase, which catalyzes the condensation of oxaloacetate and acetyl-coenzyme A in the Krebs cycle was also detectable but markedly lower in embryonic hearts compared to adult hearts (Figure 6F), indicating the Krebs cycle is able to generate FADH_2_ and NADH, which could potentially be used for electron transport.

In summary, measurements of oxygen consumption and ETC complex activity demonstrate that while ETC is inactive at E9.5, Cx-1 and Cx-2 are present and can accept electrons from an active Krebs cycle. By E11.5, Cx-2, contributes to total ETC function (oxygen consumption), although Cx-1 does have the ability to pass electrons to Cx-3. By the end of the embryonic period (E13.5), the ETC is fully functional and comparable to the adult heart.

### Formation of respirasomes coincides with the activation of electron transport

Next, we sought to determine mechanisms that account for the increase in ETC activity that occurs as the heart develops. We concentrated on the role of Cx-1 for the following reasons: 1. Entry of electrons at Cx-1 provides the most efficient generation of Δµ_H_, 2. Cx-1 plays a central role in ETC assembly [30], 3. Cx-1 activity changes dramatically during cardiac development and 4. Our initial data (Figure 6) suggests that before E13.5, Cx-1 may be able to accept electrons but does not pass them to the rest of the ETC.

#### Complex 1 deactivation and activation

**Figure 7:**
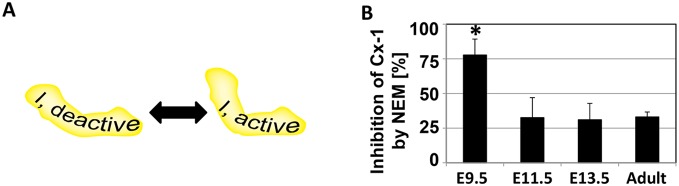
At E9.5, Cx-1 is present in its deactive form. **A.** The conversion between active and deactive Cx-1 is presumed to be related to structural changes in the multiprotein complex, and oxidation of ND3 leads to permanent deactivation. **B.** Percent of Cx-1 in deactive state, as measured by the % of NADH oxidase activity that is inhibited by NEM. *p≤0.05 compared to all other ages.

Under conditions of stress or hypoxia, Cx-1 can enter a state of deactivation, in which electron flow to ubiquinone is inhibited. This can be reversed if low concentrations of NADH are supplied, but deactivation also exposes a cysteine on the Cx-1 subunit ND3 that can be oxidized to cause irreversible deactivation [31] (Figure 7A). Our data suggests that Cx-1 may be deactivated in the early heart: the dipstick assay and NADH oxidase activity assays revealed significant activities at E9.5 (Figure 6A and B), yet reduction of ubiquinone and cytochrome *c* was not measurable. To further investigate the possibility that Cx-1 is deactive at E9.5, we incubated cardiac tissue homogenates with 1 mM N-ethylmaleimide (NEM) and measured the total NADH oxidase activity using the NADH-ferricyanide oxidoreductase assay [32]. Activity was measured before and after incubation with NEM. In the presence of NEM, more than 80% of the Cx-1 activity is lost at E9.5, while only about 30% of Cx-1 activity is lost in hearts from older embryos or adult mice (Figure 7B). These data show that Cx-1 becomes activated at ≈E11.5, the same window of time where coupling of mitochondrial oxygen consumption to oxidative phosphorylation occurs.

#### Assembly of complex 1

**Figure 8:**
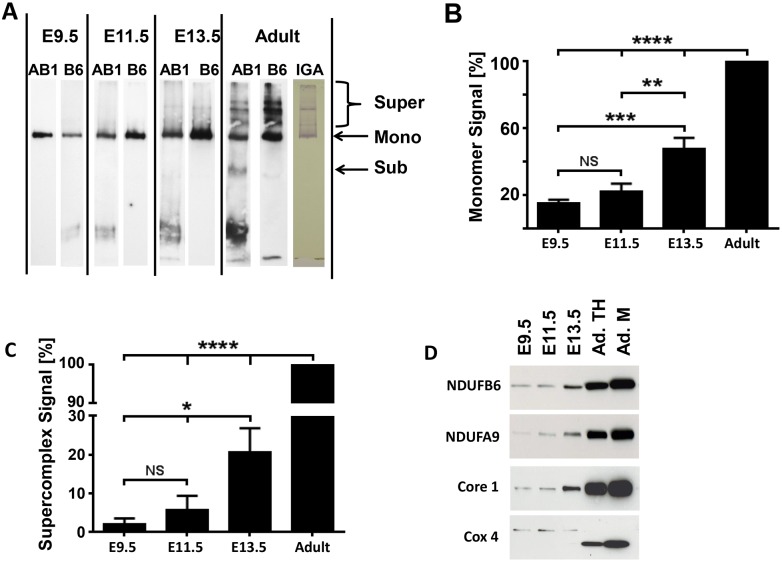
The assembly of ETC complexes into supercomplexes begins around E11.5. **A.** One-dimensional high resolution clear native PAGE (hrCN-PAGE) shows the presence of monomeric Cx-1 and the appearance of Cx-1 containing supercomplexes during embryonic development and in adult mouse hearts. Whole tissue homogenates (10 µg for embryonic samples and 5 µg for adult sample) from hearts were solubilized with digitonin (4g digitonin/g protein) and separated by 5–15% hrCN-PAGE. The proteins were then transferred onto nitrocellulose membranes and Cx-1 was visualized first by the detection of NDUFAB1 (AB1, a rabbit polyclonal antibody). Then, the membrane was stripped and re-probed against NDUFB6 (B6, a mouse-monoclonal antibody). In addition, an in-gel assay of Cx-1 (IGA) demonstrates functional complex I monomers and supercomplexes are present in adult heart homogenates. **B and C.** Quantitative analysis of the presence of monomeric Cx-1 (B) and supercomplexes containing Cx-1 (C). NS–not significant, *p≤0.05, **p≤0.01, ***p≤0.005, and ****p≤0.0001 compared to adult heart samples using ANOVA with Tukey post hoc testing; n = 5. **D.** Immunocapture of supercomplexes using anti-Cx-3 antibodies; the immunoprecipitate was analyzed by SDS PAGE/immunoblotting for the presence of subunits of Cx-1 (NDUFB6 and NDUFA9), Cx-3 (ubiquinol-cytochrome reductase core protein 1 precursor, Core 1) and Cx-4 (cytochrome c oxidase, subunit 4 (Cox 4)). Homogenates from E9.5, E11.5, and 13.5 heart tissue and homogenate (TH) and mitochondria (M) from adult hearts were examined. Representative immunoblots from n = 3, although these immunoblots are overexposed to demonstrate labeling at younger ages.

**Figure 9:**
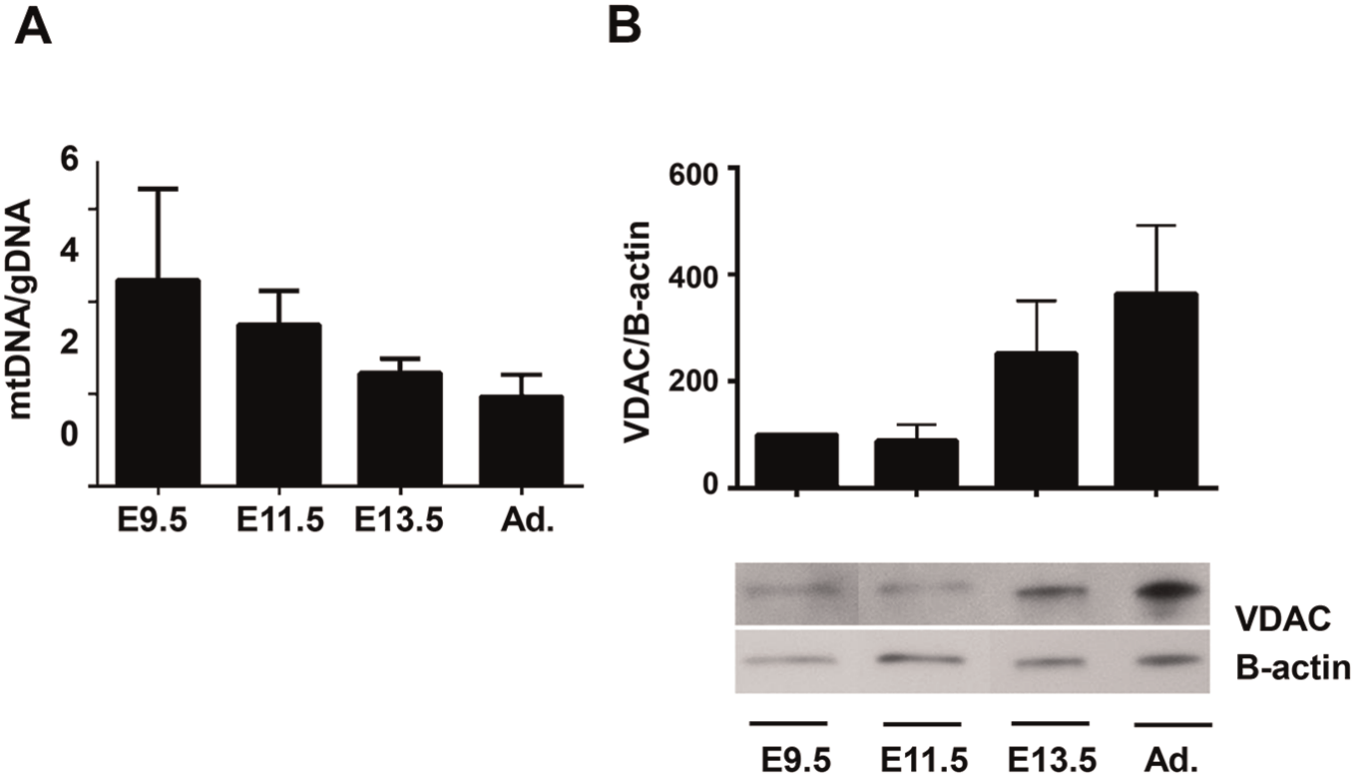
Mitochondrial biogenesis and mass measurements. **A.** The ratio of mtDNA to gDNA, a measure of mitochondrial biogenesis, was measured in samples of E9.5, 11.5, 13.5 and adult hearts using qPCR to measure levels of CO1 (mtDNA) and 18s (gDNA). **B.** (Upper) Relative expression of VDAC and beta-actin were evaluated by densitometry and normalized to the value at E9.5 (100%). No significance was determined by ANOVA, n = 4. (Lower) Representative immunoblot demonstrating VDAC and beta-actin expression at each age. Note that lanes, which are not relevant to this manuscript, between E9.5 and E11.5 have been removed.

Cx-1 activity could be decreased in the early embryonic heart if it is not assembled in significant amounts to perform its function. To determine if Cx-1 monomers are present, we performed hrCN PAGE and immunolabeled samples for two Cx-1 subunits: NDUFB6 and NDUFAB1, which reside in the membranous and peripheral arms of Cx-1, respectively. Immunoblots demonstrate that the 880 kDa Cx-1 monomer is fully assembled in the embryonic heart at E9.5 (Figure 8A). Quantification revealed that E13.5 heart homogenates contained about half of the monomers observed in the adult heart, perhaps due to the increased mitochondrial volume in the latter (Figure 8B, Figure 9). Furthermore, E9.5 and 11.5 had equally less monomers compared to older samples (Figure 8B).

#### The formation of respiratory supercomplexes

To increase the efficiency of the ETC in some tissues including the heart, Cx-1 is thought to form “solid state” supercomplexes with Cx-3, Cx-4, ubiquinone, and cytochrome *c* [8], [10], [30] Therefore, as our data suggests that the efficiency of electron transfer from Cx-1 to Cx-3 increased with embryonic age, we investigated whether assembly of respiratory supercomplexes increases during cardiac development.

First, we analyzed hrCN PAGE immunoblots for the presence of supercomplexes containing Cx-1 (Figure 8A). It has been reported that C57BL/6J mice do not assemble supercomplexes [9]. However, the heart homogenates from C57BL/6N adult mice used in these studies do assemble complex I-containing supercomplexes based on immunoblotting and enzyme activity (Figure 8A, Adult). In contrast, quantification revealed that Cx-1-containing supercomplexes were virtually absent in E9.5 samples. However, as the embryos grew older and the mitochondria matured, supercomplexes containing Cx-1 became apparent: there were slightly more at E11.5 and significantly more at E13.5, respectively, although levels did not approach those seen in adult homogenates (Figure 8A and C).

To substantiate these results, we determined whether Cx-1 and Cx-4 could be co-precipitated with a Cx-3 immunocapture antibody from cardiac homogenates (Figure 8D). First, we verified immunoprecipitation of Cx-3 by labeling the precipitate against a different Cx-3 subunit, the ubiquinol-cytochrome reductase core protein 1 precursor (Figure 8D, Core 1). When we labeled the precipitate against Cx-1 subunits, and found that NDUFB6 was detectable throughout embryonic cardiac development in all precipitates, while the NDUFA9 was barely detectable in E9.5 hearts but was seen in increasing amounts in older hearts (Figure 8D, NDUFB6 and NDUFA9). We also found that the Cx-4 subunit IV (Cox4) was detectable in embryonic hearts. However, the most dominant band in the precipitates from adult hearts had, as predicted, a molecular weight of 17 kD, while the band labeled in embryonic precipitates had a molecular weight of about 20 kD (Figure 8D), which could be due to a switch in COX4 isoforms or in precursor processing [33]. Finally, we note that it is possible that our protocol could independently precipitate protein aggregates that do not contain all components of the supercomplex. However, the combination of our immunoblotting and in gel assay (Figure 8A) and these immunoprecipitation experiments suggest that fully formed supercomplexes are present in older embryonic and adult hearts.

### Mitochondrial mass and biogenesis during embryonic cardiac development

Finally, we examined changes in mitochondrial biogenesis during cardiac development, as this could affect effect interpretation of our results. To examine mitochondrial biogenesis we performed quantitative PCR of mitochondrial (mt) and genomic (g) DNA and found that the mtDNA/gDNA ratio steadily increased from E9.5 to adult hearts (Figure 9A). Next, we quantified the expression of the mitochondrial outer membrane protein VDAC and found that E9.5 and 11.5 hearts had lower levels of this protein than E13.5 and adult hearts (Figure 9B). However, there were no statistically significant differences between the ages in either experiment. In contrast, in our previous work, we noted that, in cells cultured from E9.5, 11.5, and 13.5 hearts, the area occupied by mitochondria significantly increased between E9.5 and 13.5 (published as supplemental Figure S1C in [6]). Overall, these data suggest a decrease of mitochondrial biogenesis and an increase of mitochondrial mass during cardiac development.

## Discussion

We previously demonstrated that embryonic cardiac development is associated with a maturation of mitochondrial structure and an increase in Δψ_m_, which result from closure of the mPTP after E9.5 and before E13.5. The closure of the mPTP in the embryonic heart also reduces oxidative stress and enhances myocyte differentiation [6]. The results presented here show that the ETC activity is essentially non-functional in the early embryo, but increases as the heart develops so that by E13.5 its function is comparable to that observed in adult hearts. We also describe the assembly of respiratory supercomplexes and activation of Cx-1 as important mechanisms which lead to an increase of ETC activity.

### ETC activity increases during cardiac development

It is logical to assume that energy production must increase as the heart develops to ensure survival. In the early mammalian embryo, oxygen diffuses from maternal tissues, oxygen tension is thought to be low, and aerobic glycolysis supplies ATP to support embryonic survival. However, in the mouse, growth of the embryo dictates establishment of the placenta and a functional cardiovascular system after about E10 (reviewed in [1]–[3]). It is also reasonable to assume that mitochondrial OXPHOS, which depends upon oxygen as the final electron acceptor, may be limited by the physiologic hypoxia in the early embryo but that, after E10, OXPHOS activity can commence and ATP production will increase to supply energy to the heart as its output must increase to accommodate embryonic growth.

The changes we report here are consistent with this increase in OXPHOS activity after E10. Some of this increased activity can be accounted for by the apparent increase in mitochondrial biogenesis followed by an increase in mitochondrial mass during the embryonic period (Figure 9). However, a dramatic increase in the function of individual mitochondria probably accounts for much of this change in OXPHOS activity. Although a few older studies suggest an increase in mitochondrial function during the embryonic and fetal period (the “fetal switch;” reviewed in [1], [4]), it has been generally, but incorrectly, reported that the switch to aerobic metabolism occurs after birth. The role of bioenergetics during early cardiac development has received little investigation over the last two decades as studies have concentrated on the complex intra- and extra-cellular signaling networks that control gene expression. In addition, a detailed examination of mitochondrial function is difficult in small samples such as those obtained from the embryonic heart. However, we overcame this limitation by adapting standard mitochondrial techniques and using cardiac tissue homogenates to measure mitochondrial oxidative activity. The use of tissue homogenate or permeabilized cells to assess oxidative function has been described previously [15], [34]. Although we acknowledge that the presence of extra-mitochondrial proteins in tissue homogenates may affect some of our results (e.g., Figures 3 and 4, E13.5 and adult), we believe that comparing results between different embryonic ages describe real changes in ETC function. In addition, the combined data from multiple assays (Clark electrode, dipstick and activity assays) and preparations (tissue homogenates and purified mitochondria) support our conclusions regarding changes in ETC function that occur during embryonic heart development. Furthermore, a different approach, where maintaining a heart rate using anaerobic and aerobic substrates in early embryonic rats was investigated, support our results [35].

Finally, we recognize that these data do not represent the entire period of cardiac development. We describe a transition in ETC function at about E11.5, so examining E10.5 and E12.5 hearts would be informative, especially if specimens from fetal and neonatal mice were included to determine the changes that occur during these critical periods of development. More work must be done to address these issues.

### Increased efficiency of electron transport activity after E9.5

Our data show that cardiac mitochondria at E9.5 are uncoupled, exhibit minimal ETC activity, and that both coupling (discussed below) and ETC activity increase as the heart develops. This increase in ETC activity is accompanied by increases in electron transfer by Cx-1 and Cx-2 and by the assembly of respiratory supercomplexes. Although we did not measure ATP production directly, others have shown this to increase in parallel to the changes in the ETC that we have observed (Baker CN and Ebert SN, Weinstein Cardiovascular Development Conference, 2013).

Electrons can enter the ETC via a number of sources, but the major routes are through Cx-1 and Cx-2, which accept electrons from NADH and FADH_2_, respectively. Since Cx-1, 3, and 4 pump protons from the matrix to the inter membrane space, electron entry via Cx-1 is more efficient to generate Δµ_H_ than entry via Cx-2. Our data show that activation of Cx-1 lags behind that of Cx-2 by a day or two during early mouse cardiac development. Perhaps this is a protective mechanism, in that early activation of Cx-1 might inappropriately increase the need for oxygen consumption when oxygen concentrations are still low. The earlier activation of Cx-2 might allow for some ETC activity with less reduction of oxygen while also allowing the Krebs cycle to stay active to provide metabolic intermediates for other biosynthetic processes [36].

Our data suggest that one mechanism by which ETC efficiency increases is the activation of Cx-1. Cx-1 is a mammoth structure containing 43–45 subunits [37], [38], so one might speculate that its inactivity is due to its lack of assembly. However, immunoblots demonstrate significant levels of Cx-1 monomers at all embryonic ages (Figure 8B and C). De-activation of Cx-1 in the absence of oxygen is an intrinsic property of this enzyme [31] and we find that about 80% of Cx-1 are in the de-active form at E9.5 (Figure 7), a time when the embryo is in a physiologic hypoxic environment. Perhaps with the establishment of placental and cardiovascular function, subsequent increases in oxygen levels after E10 drive Cx-1 activation and increased ETC activity in the developing embryonic heart. Alternatively, it is also suggested that lack of electron flux through Cx-1 to the rest of the ETC causes deactivation [39], and our data indicate that this state exists at E9.5. Finally, recent reports demonstrate that NDUFA9 is a Cx-1 assembly factor that binds to the ubiquinone binding site [40], [41]. Therefore, the increased association of the NDUFA9 subunit to the supercomplex later in development (Figure 8C) could also be related to the observed increase in Cx-1 activation.

Convincing data from a number of laboratories [9], [10], [30], [42] demonstrate that the two models of the ETC, the fluid (random collision) model and the solid-state (supercomplex) model, are not mutually exclusive. In fact, the “plasticity model” of the ETC allows for transition between fluid and solid ETC states depending upon the conditions faced by the cell [42]. Our data suggest that the lack of ETC activity in the early embryonic heart may also be due, in part, to a transition from a fluid to a more solid ETC state after E11.5 due to increased respiratory supercomplex formation. This would increase the efficiency of electron flux between Cx-1, 3, and 4. Our data show the absence of supercomplexes before E11.5 despite the presence of Cx-1 monomers, which are required for supercomplex assembly [11]. In addition, since the activation state of Cx-1 does not dictate its assembly into supercomplexes [40], deactive Cx-1 at E9.5 alone cannot explain the absence of supercomplex formation at this developmental stage.

Our data demonstrate that respiratory supercomplexes do form in hearts from C57BL/6N mice (Figure 8A), in contrast to a recent report [9]. However, we used C57BL/6J mice, which are known to have at least one mutation that affects mitochondrial function–the nicotinamide nucleoside transhydrogenase (*Nnt*) gene is inactivated [43]. It is also possible that our use of hrCN PAGE and cardiac tissue, as opposed to Blue Native PAGE and liver tissue or fibroblasts as in [9], could account for the observed differences. Further evaluation will have to be performed to determine the reason for and significance of these two differing results.

All of these mechanisms may help explain the developmental changes we see in oxygen consumption, although future experiments are needed to determine the molecular mechanisms controlling the composition and stoichiometry of respirasomes during embryonic development.

### Implications of increased ETC activity during cardiac development

Additional studies support the critical importance of ETC activation during cardiac development. First, a number of transgenic animal models demonstrate that dysfunctional or missing components of the ETC, including *Cytc*, *Ndufa13*, *Ndufs4*, and *Sdhd*, lead to embryonic demise [4], [5], [44]. Second, a number of mouse models that exhibit mid-embryonic demise from heart failure exhibit abnormal cardiac mitochondria and mitochondrial dysfunction, including mice lacking *Dbh*, *Nfactc3/Nfatc4*, *Rxra*, and *Slc8a1* [4], [45]–[47]. The death observed in all of these models occurs precisely when electron transport becomes more active and inhibitors of the ETC (rotenone, malonate, and KCN) become more specific in our experiments, suggesting that ETC activation in the early embryonic heart is a critical mechanism that ensures survival during development. We speculate that analysis of embryonic cardiac function by *in utero* echocardiography would reveal systolic dysfunction in these transgenic models, but to the best of our knowledge such studies have not been attempted.

Functional data presented here indicate that in the mouse ∼E11.5 is a critical period for ETC activity. First, we show a decrease in V_0_ at this stage of development, resulting in an increase of the RCR (indicating coupling of ETC activity and mitochondrial ATP synthesis). Second, the variable effect of ATR on oxygen consumption supports the hypothesis that the critical change ETC activity occurs at E11.5 (Figure 5). It is important to point out that ATR is not only an inhibitor of the ETC but is also used to induce mPTP activation [28], [48], which would uncouple respiration. These current data, which show either inhibition or uncoupling of ETC activity by ATR, suggest that a change in the regulation of the mPTP occurs around E11.5 and are consistent with our previous report of mPTP function in the embryonic heart [6]. We recently defined the c-ring of the F_0_ subunit of the ATP synthase as mPTP [49] and future experiments using this knowledge may help to identify molecular components altered ∼E11.5, which are responsible for reducing the probability of mPTP opening and the begin of ETC activity.

**Figure 10:**
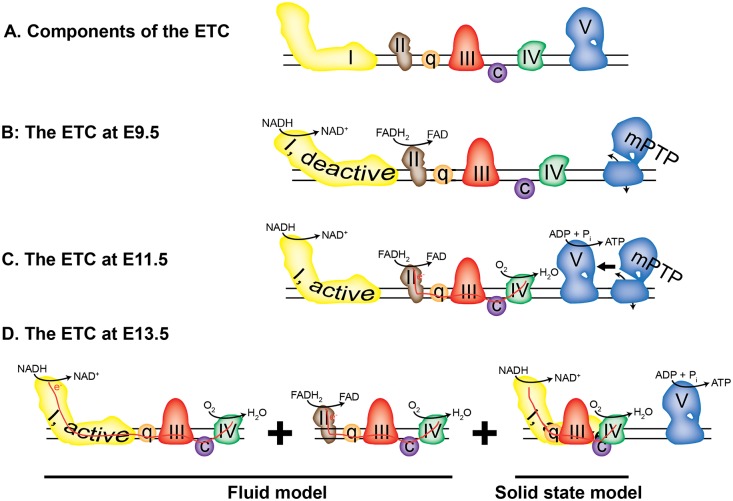
Model of the embryonic heart ETC. **A.** Components of the ETC; the complexes are labeled with Roman numerals. **B.** At E9.5, the complexes are arranged randomly and do not participate in electron transport, although Cx-1 and -2 have NADH and FADH_2_ oxidase activity. The mPTP, represented by a not-fully-assembled Cx-5, is open. **C.** At E11.5, Cx-2, but not Cx-1, participates in electron transport (red line) and oxygen consumption. The mPTP is open or closed. **D.** At E13.5, the ETC exists in both the fluid (left two panels) and solid (right panel) assembly states, while the mPTP is closed.

Finally, our results may also explain changes in mitochondrial ultrastructural complexity previously observed in the developing heart [6]. Mitochondrial morphology and the functionality of the ETC are intertwined, as the assembly of supercomplexes drives the formation of the cristae within the IMM [50], [51]. Thus, the absence of respiratory supercomplexes may explain the simplified structure that we observed in E9.5 hearts and the increased assembly of supercomplexes and/or closure of the mPTP during cardiac development correlates with this increased complexity of the IMM. In conclusion, our data indirectly confirm that the early embryo relies upon anaerobic glycolysis for energy and that OXPHOS is fully active by the end of the embryonic period (Figure 10).
